# Association between elevated multiple circulating biomarkers and short-term mortality in critically ill lung cancer patients: a meta-analysis

**DOI:** 10.3389/fonc.2026.1861784

**Published:** 2026-08-19

**Authors:** Congcong Li, Jing Miao, Liqing Gao, Dingwen Zheng

**Affiliations:** 1 Department of Critical Care Medicine, Sir Run Run Shaw Hospital, School of Medicine, Zhejiang University, Hangzhou, Zhejiang, China; 2 Department of Cardiac Surgery, Sir Run Run Shaw Hospital, School of Medicine, Zhejiang University, Hangzhou, Zhejiang, China

**Keywords:** critical care, lung cancer, marker, meta-analysis, prognosis, short-term mortality

## Abstract

**Background and aims:**

While traditional circulating biomarkers have demonstrated prognostic value in general lung cancer patients, their short-term predictive value in critical care settings remains unclear. This study aims to systematically evaluate the association between elevated multiple circulating biomarkers and short-term mortality in critically ill lung cancer patients.

**Methods:**

This study searched databases including PubMed, Embase, Web of Science, Cochrane Library, and CNKI from inception to February 2026. Literatures that enrolled patients with severe lung cancer, reported data on the association between biomarkers and short-term mortality, and provided relative risks (RR) or convertible data were included. Meta-analysis was conducted using R software with the Hartung-Knapp-Sidik-Jonkman (HKSJ) method for random effects models and robust variance estimation (RVE) to handle correlated effect sizes from the same study. Heterogeneity was assessed, and subgroup analyses, meta-regression, and sensitivity analyses were performed to evaluate pooled effect sizes and publication bias.

**Results:**

This study included 9 literatures, extracting 19 studies covering 4,436 critically ill lung cancer patients. The primary meta-analysis using RVE showed that elevated biomarkers were significantly associated with short-term mortality (pooled RR = 1.62, 95% CI: 1.09-2.41, P = 0.022). Among these, 17 studies showed positive associations, with pooled RR = 1.93 (95% CI: 1.50-2.48, P < 0.001). Subgroup analysis revealed significant differences in effect sizes across different outcomes, biomarker types, and geographical regions (all P < 0.01 between subgroups), with the inflammatory biomarker subgroup showing the highest effect size (RR = 2.44). Subgroup analysis by critical illness definition showed that the effect was significant in both ICU-admitted patients (RR = 1.38, 95% CI: 1.07-1.78) and patients with advanced/terminal disease (RR = 2.45, 95% CI: 1.66-3.62). Meta-regression analysis indicated that outcome measures (90-day mortality) and patient region (North America) were significant factors influencing heterogeneity. Sensitivity analysis confirmed the robustness of results.

**Conclusion:**

Multi-biomarker detection provides important reference for risk stratification and treatment decision-making in critically ill lung cancer patients. Future clinical practice could combine traditional critical care scoring systems to establish individualized prognostic prediction models based on multiple biomarkers.

## 1 Introduction

Lung cancer is one of the malignant tumors with the highest incidence and mortality rates worldwide. Critically ill lung cancer patients, particularly those requiring intensive care, face extremely high mortality risks and complex clinical management challenges (1). Accurate assessment of short-term prognosis for these patients is crucial for developing rational treatment strategies, optimizing resource allocation, and improving physician-patient communication (2). Prognostic assessment for critically ill lung cancer patients faces unique challenges: first, cancer-related cachexia, immunosuppression, and systemic inflammatory responses (3); second, organ toxicity related to anti-tumor therapy; third, multiple organ dysfunction associated with critical illness; and fourth, complex physician-patient decision-making and ethical considerations.

Traditional critical care scoring systems such as APACHE II and SOFA scores play important roles in prognostic assessment for critically ill patients, but these systems are primarily based on physiological parameters and organ function (4), with relatively insufficient consideration of cancer-specific factors. Additionally, these scoring systems require multiple parameters and complex calculations, which may not be readily available during emergency or early critical care phases.

Circulating biomarkers, as important indicators reflecting tumor biological behavior, play crucial roles in lung cancer diagnosis, treatment monitoring, and prognostic assessment. In this study, we define “circulating biomarkers” broadly to include a range of blood-based laboratory parameters that have demonstrated prognostic value. These markers have different expression patterns and clinical significance in different pathological types of lung cancer (5–7). In recent years, increasing numbers of novel markers have been discovered, including: (1) inflammation-related markers such as CRP, NLR, PLR, IL-6 (3); (2) metabolism-related markers such as lactate, albumin, lactate/albumin ratio (8, 9); (3) enzymatic markers such as LDH, AST, ALP (10); (4) organ function markers such as bilirubin, BUN, creatinine (11).

Recently, increasing research has focused on the prognostic value of biomarkers in critically ill cancer patients. Studies have shown that inflammatory markers such as NLR and PLR demonstrate good predictive value in critically ill tumor patients (12). Research has found that lactate/albumin ratio outperforms traditional scoring systems in mechanically ventilated patients (13). LDH is an important prognostic factor in immunotherapy patients (14). These findings provide new insights for prognostic assessment in critically ill lung cancer patients.

Therefore, this study uses meta-analysis methodology to systematically evaluate the association between elevated multiple markers and short-term mortality in critically ill lung cancer patients and explore sources of heterogeneity, providing evidence-based medical evidence for clinical practice.

## 2 Materials and methods

### 2.1 Literature search strategy

This study followed PRISMA guidelines (15) for systematic review and meta-analysis. Systematic searches were conducted in PubMed, Embase, Web of Science, Cochrane Library, and China National Knowledge Infrastructure (CNKI) databases from inception to February 2026. The search strategy combined subject terms with free text terms, including: lung cancer, lung neoplasm, pulmonary carcinoma, intensive care, critical care, ICU, critically ill, tumor marker, biomarker, CEA, CYFRA21-1, NSE, CA125, carcinoembryonic antigen, mortality, death, survival, prognosis, short-term, 30-day, in-hospital, early. Detailed search strategies for each database are shown in **Supplementary Table 1**. References of relevant literatures were also searched to supplement missed studies.

### 2.2 Inclusion and exclusion criteria

Inclusion criteria: (1) Study subjects were pathologically confirmed lung cancer patients, including NSCLC and SCLC; (2) Patients were in critical condition, requiring ICU treatment, APACHE II score ≥15, SOFA score ≥6, or having other critical characteristics; (3) Reported relationship between markers and short-term mortality, with short-term defined as ≤180 days; (4) Provided RR, HR, OR with corresponding 95% confidence intervals (CI), or convertible data; (5) Study design was prospective or retrospective cohort study, case-control study; (6) Published in Chinese or English.

Exclusion criteria: (1) Case reports, reviews, commentaries, conference abstracts; (2) Incomplete data or inability to extract effect sizes; (3) Duplicate publications; (4) Animal experiments or *in vitro* studies; (5) Studies with sample size <30 cases.

### 2.3 Data extraction and quality assessment

The following information was extracted from each retrieved literature: (1) Basic information: first author, publication year, study country, study design; (2) Study subjects: sample size, patient age, gender, lung cancer type, TNM stage; (3) Critical illness definition: ICU admission, end-stage, APACHE II score, SOFA score, etc.; (4) Biomarkers: biomarker type, detection method, cutoff value, abnormal rate; (5) Follow-up information: follow-up time, loss to follow-up rate; (6) Outcome measures: short-term mortality, effect size (RR or OR or HR) and 95% CI. Two researchers independently extracted data, with disagreements resolved by a third researcher.

The Newcastle-Ottawa Scale (NOS) was used to evaluate cohort study quality, assessing selection, comparability, and outcome dimensions, with a total score of 9 points. Scores of 0-3, 4-6, and 7–9 were considered low, moderate, and high quality, respectively. The NOS scores for each study are reported in **Supplementary Table 2**.

### 2.4 Statistical analysis

Meta-analysis was conducted using the R software “meta” package for meta-analysis, publication bias testing, and sensitivity analysis, with the “metafor” package (16) used for meta-regression analysis and the “robumeta” package for robust variance estimation (RVE). RR and its 95% CI were used as effect size indicators. For studies providing OR or HR, standard conversion methods were used: ORs were converted to RR using the formula RR = OR/((1 - P0) + (P0 × OR)), where P0 was the baseline risk estimated from the control group; HRs were approximated as RR under the proportional hazard’s assumption. The primary analysis used RVE with correlated effects to account for multiple biomarkers from the same study. Sensitivity analyses using the DerSimonian-Laird random effects method were also performed to confirm the robustness of findings. For the primary RVE analysis, the Hartung-Knapp-Sidik-Jonkman (HKSJ) method was used to calculate pooled RR and its 95% CI. Cochran’s Q test and I² statistic were used to assess heterogeneity between studies: I² <25% indicated low heterogeneity, 25-50% moderate heterogeneity, and >50% high heterogeneity. Given the expected clinical and methodological diversity, random effects models were used *a priori*.

Sources of heterogeneity were explored through: subgroup analysis by different outcome measures (ICU mortality, In-hospital mortality, 14-day mortality, 90-day mortality, and 180-day mortality), biomarker types (Inflammatory biomarker, Metabolic biomarker, Enzymatic biomarker, and Organ functional biomarker), patient geographical regions (East Asia, North America, South America, and Middle East), and critical illness definition (ICU admission vs. advanced/terminal disease); meta-regression analysis to explore the influence of sample size, different outcomes, biomarker types, and patient geographical regions on heterogeneity.

Sensitivity analysis was conducted by sequentially excluding each study and recalculating pooled effect sizes to assess result stability. Egger’s test and Begg’s test were used to assess publication bias, with *P* < 0.05 indicating significant publication bias. If publication bias existed, trim-and-fill method was used to adjust effect sizes. All statistical tests were two-sided, with *P* < 0.05 considered statistically significant.

## 3 Results

### 3.1 Literature screening results

**Figure 1:**
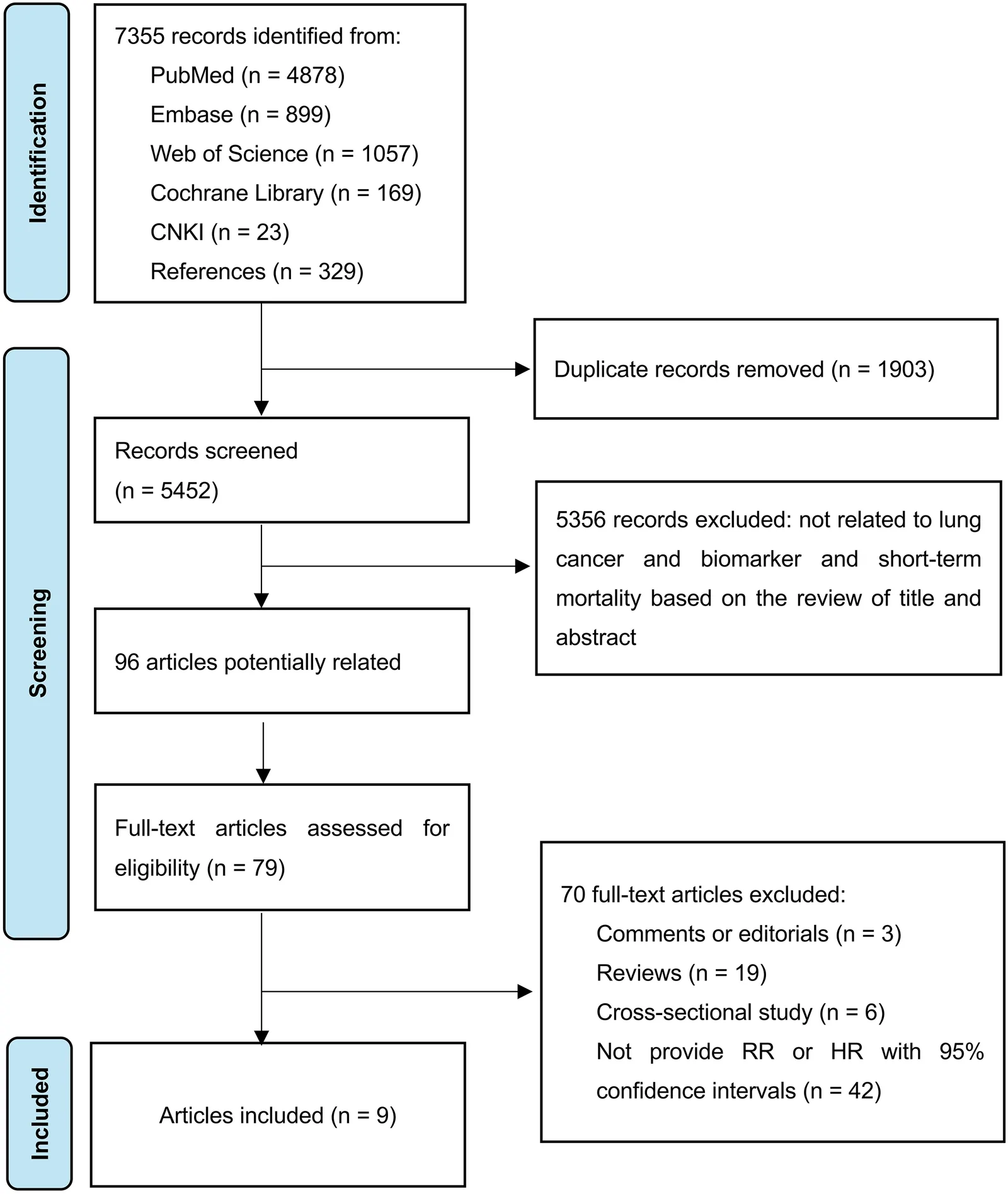
PRISMA flow diagram of the literature search and selection.

Using the search strategy (see **Supplementary Table 1**), this study retrieved 7,026 literatures from PubMed, Embase, Web of Science, Cochrane Library, and China National Knowledge Infrastructure (CNKI) databases, plus an additional 329 literatures through reference searching, totaling 7,355 literatures. After deduplication, 5,452 literatures remained. Applying inclusion and exclusion criteria, 96 literatures remained after screening titles and abstracts. After full-text evaluation, 9 literatures (17–25) were finally included, covering 4,436 critically ill lung cancer patients. The literature screening process, from initial search to final included studies, including the number of literatures and exclusion reasons at each stage, is shown in **Figure 1**.

### 3.2 Basic characteristics of included studies

In this study, some literatures contained multiple different markers (18, 19, 22, 25), with each biomarker having different effect sizes on short-term mortality. Therefore, in this meta-analysis, multiple biomarkers from the same study are correlated, we employed robust variance estimation (RVE) in our primary analysis to account for this dependency. For descriptive purposes and secondary analyses, each biomarker from these literatures was treated as an independent study. This study extracted a total of 19 studies from 9 literatures. Study and patient characteristics are shown in **Table 1**. This study used the Newcastle-Ottawa Scale (NOS) to assess the quality of included literatures. NOS scale scores for all included literatures are shown in **Supplementary Table 2**.

**Table 1:** Basic characteristics of all included studies.

Study	Country	Study design	Cases	Male/female (*n*)	Type of patients	Definition of critical illness	Region of patients	Biomarker	Biomarker type	Outcome	NOS score
Al-Dorzi, 2024 (17)	Saudi Arabia	Retrospective study	306	209/97	ICU patients	ICU admission	Middle East	Serum lactate	Metabolic biomarker	In-hospital mortality	8
Fang, 2025a (18)	China	Retrospective study	645	331/314	ICU patients	ICU admission	North America*	Alkaline phosphatase (ALP)	Enzymatic biomarker	In-hospital mortality	8
Fang, 2025b (18)	China	Retrospective study	645	331/314	ICU patients	ICU admission	North America*	Bilirubin total	Hepatic/functional biomarker	In-hospital mortality	8
Long, 2025 (20)	China	Retrospective study	154	112/42	ICU patients	ICU admission	East Asia	Serum lactate	Metabolic biomarker	ICU mortality	7
Peixoto, 2022 (21)	Brazil	Retrospective study	110	56/54	ICU patients	ICU admission	South America	C-reactive protein (CRP)	Inflammatory biomarker	In-hospital mortality	7
Tanaka, 2022a (25)	Japan	Retrospective study	649	465/184	Terminal patients	Advanced lung cancer	East Asia	C-reactive protein (CRP)	Inflammatory biomarker	14-day mortality	8
Tanaka, 2022b (25)	Japan	Retrospective study	649	465/184	Terminal patients	Advanced lung cancer	East Asia	Aspartate aminotransferase (AST)	Enzymatic biomarker	14-day mortality	8
Tanaka, 2022c (25)	Japan	Retrospective study	649	465/184	Terminal patients	Advanced lung cancer	East Asia	Blood Urea Nitrogen (BUN)	Renal/functional biomarker	14-day mortality	8
Tanaka, 2022d (25)	Japan	Retrospective study	649	465/184	Terminal patients	Advanced lung cancer	East Asia	White Blood Count (WBC)	Hematologic/inflammatory biomarker	14-day mortality	8
Tanaka, 2022e (25)	Japan	Retrospective study	649	465/184	Terminal patients	Advanced lung cancer	East Asia	Neutrophil-to-Lymphocyte Ratio (NLR)	Hematologic/inflammatory biomarker	14-day mortality	8
Tanaka, 2022f (25)	Japan	Retrospective study	649	465/184	Terminal patients	Advanced lung cancer	East Asia	Red cell Distribution Width (RDW)	Hematologic/inflammatory biomarker	14-day mortality	8
Wang, 2025a (19)	China	Retrospective study	455	317/138	Terminal patients	Advanced lung cancer	East Asia	C-reactive protein (CRP)	Inflammatory biomarker	90-day mortality	8
Wang, 2025b (19)	China	Retrospective study	455	317/138	Terminal patients	Advanced lung cancer	East Asia	Interleukin-6 (IL-6)	Inflammatory biomarker	90-day mortality	8
Wang, 2025c (19)	China	Retrospective study	455	317/138	Terminal patients	Advanced lung cancer	East Asia	Serum albumin (ALB)	Nutritional/hepatic synthetic biomarker	90-day mortality	8
Wang, 2025d (19)	China	Retrospective study	455	317/138	Terminal patients	Advanced lung cancer	East Asia	Lactate dehydrogenase (LDH)	Enzymatic biomarker	90-day mortality	8
Xu, 2026 (23)	China	Retrospective study	1046	586/460	ICU patients	ICU admission	North America*	Serum lactate	Metabolic biomarker	In-hospital mortality	8
Zhao, 2019a (22)	China	Retrospective study	98	63/35	Terminal patients	Advanced lung cancer	East Asia	Serum PCNA	Cellular proliferation biomarker	180-day mortality	7
Zhao, 2019b (22)	China	Retrospective study	98	63/35	Terminal patients	Advanced lung cancer	East Asia	Serum PDCD5	Apoptotic biomarker	180-day mortality	7
Zhang, 2026 (24)	China	Retrospective study	973	485/488	ICU patients	ICU admission	North America*	Red cell distribution width-to-albumin ratio (RAR)	Inflammatory/Nutritional biomarker	180-day mortality	8

Some literatures contain multiple different biomarkers, and the effect size of each biomarker on short-term mortality is different. Therefore, each biomarker in these literatures is regarded as an independent study. A total of 19 studies were extracted from the selected 9 literatures in this study.

*: The patients in these studies were sourced from the MIMIC-IV 2.2 database, which records admissions to intensive care units (ICUs) at Beth Israel Deaconess Medical Center from 2008 to 2019, So the patients in these studies are located in North America.

Among the 19 studies, the most frequently reported outcome measure was 14-day mortality (6 studies [32%]) (25), followed by in-hospital mortality (5 studies [26%]) (17, 18, 21, 23) and 90-day mortality (4 studies [21%]) (19), then 180-day mortality (3 studies [16%]) (22, 24). Different studies also reported different biomarkers, with the most frequently reported biomarkers being C-reactive protein (CRP) (3 studies [16%]) (19, 21, 25) and serum lactate (3 studies [16%]) (17, 20, 23), with all other biomarkers reported in only 1 study [5%], reflecting the important role of inflammatory and metabolic biomarkers in prognostic assessment of critically ill lung cancer. The detailed units of measurement and cut-off values for each biomarker are provided in **Supplementary Table 3**.

### 3.3 Association between elevated markers and short-term mortality in critically ill lung cancer patients

**Figure 2:**
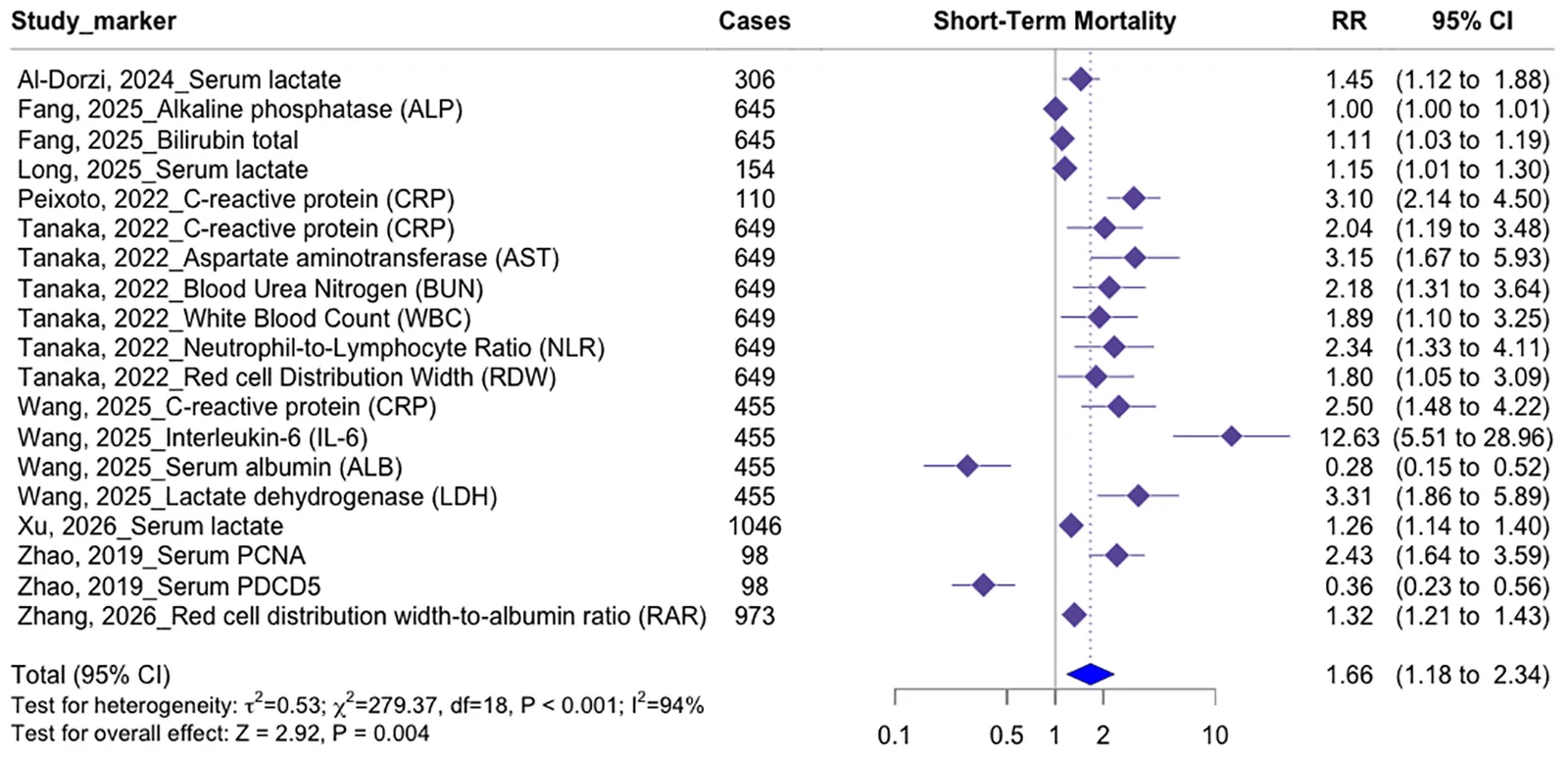
Forest plot of the association between elevated biomarkers and short-term mortality in critically ill lung cancer patients across all studies (RVE analysis).

The primary meta-analysis employed robust variance estimation (RVE) to account for the correlation between multiple biomarkers from the same study. Combining results from all 19 studies involving 4,436 patients, the overall pooled effect size was RR = 1.62 (95% CI: 1.09-2.41, P = 0.022) (**Figure 2**), indicating that elevated biomarkers in critically ill lung cancer patients significantly increase short-term mortality. The Hartung-Knapp-Sidik-Jonkman (HKSJ) method yielded consistent results (pooled RR = 1.62, 95% CI: 1.09-2.41, P = 0.036). Heterogeneity analysis revealed high heterogeneity between studies (*I²* = 94%, *P* < 0.001) (**Figure 2**). This high heterogeneity may stem from: (1) different types of biomarkers having different biological mechanisms; (2) different studies using different definitions of critical illness and outcome measures; (3) differences in geographical regions and medical levels; (4) differences in sample sizes. Given this high heterogeneity, the pooled estimate should be interpreted with caution as an average effect across a diverse set of studies.

**Figure 3:**
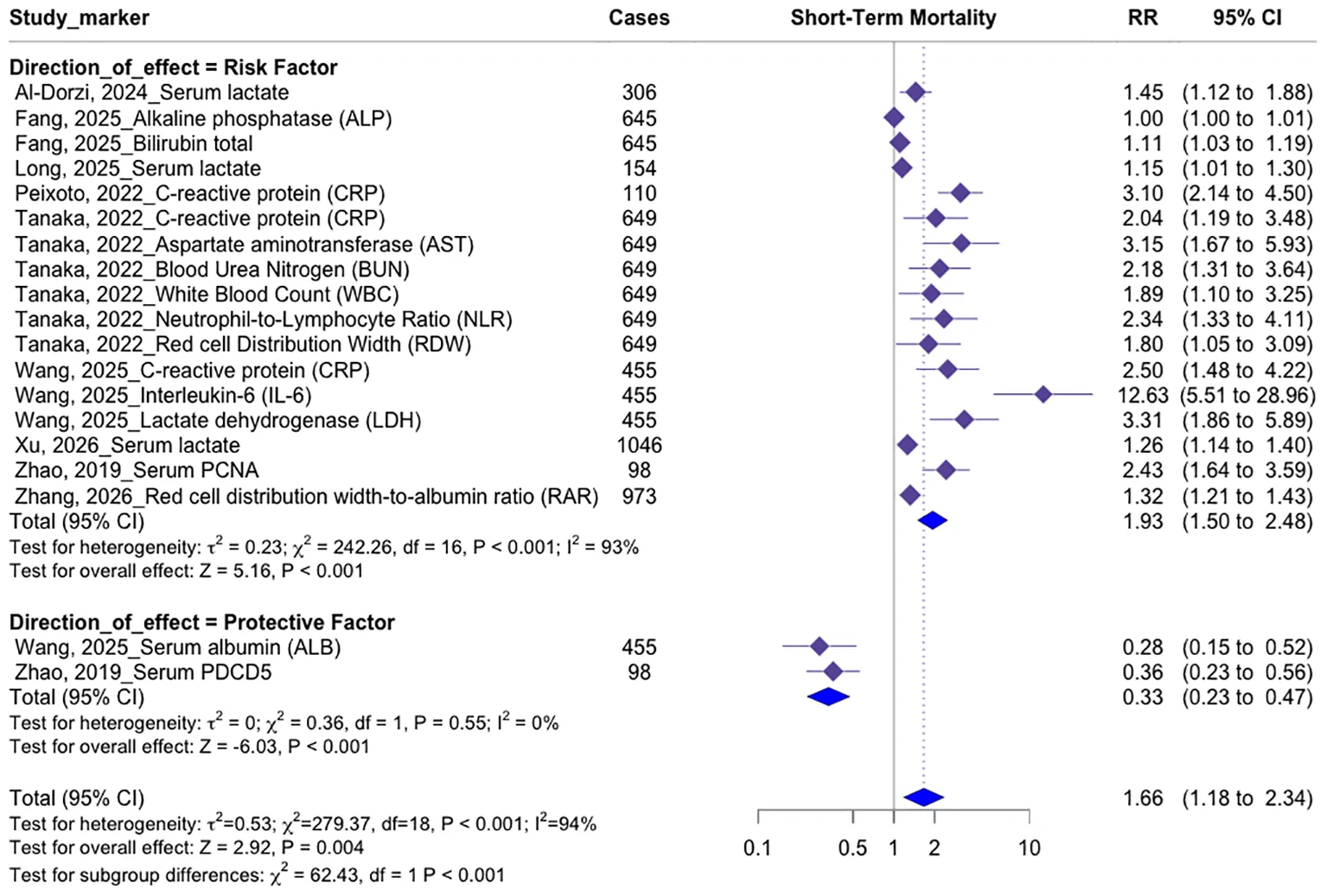
Forest plot of the association between elevated biomarkers and short-term mortality by different directions of effect.

Among all 19 included studies, the majority (17 studies) (17–25) reported significant positive associations between elevated markers and short-term mortality in critically ill lung cancer patients, with pooled RR = 1.93 (95% CI: 1.50-2.48, *P* < 0.001) (**Figure 3**), indicating that elevated markers are risk factors for short-term mortality in critically ill lung cancer patients. High heterogeneity existed between these 17 studies (*I²* = 93%, *P* < 0.001).

Additionally, among all 19 studies, 2 studies (19, 22) reported significant inverse associations between elevated biomarkers and short-term mortality in critically ill lung cancer patients, with pooled RR = 0.33 (95% CI: 0.23-0.47, *P* < 0.001) (**Figure 3**), indicating that serum albumin (ALB) and serum PDCD5 are protective factors for short-term mortality in critically ill lung cancer patients. No heterogeneity existed between these 2 studies (*I²* = 0%, *P* = 0.55).

### 3.4 Subgroup meta-analysis

Subsequently, based on the 17 studies showing significant positive associations between elevated markers and short-term mortality in critically ill lung cancer patients, this study conducted subgroup analyses by different outcome measures, different types of biomarkers, different patient geographical regions and different critical illness definitions. All subgroup analysis results are summarized in **Table 2**.

**Table 2:** Summary of subgroup analysis results.

Variables	Cases	RR (95% CI)	*P* value	*I²* (%)
Overall	4436	1.93 (1.50-2.48)	< 0.001	93
Outcome				
ICU mortality	154	1.15 (1.01-1.30)	–	–
In-hospital mortality	2107	1.40 (0.98-2.01)	0.07	94
14-day mortality	649	2.15 (1.72-2.70)	< 0.001	0
90-day mortality	455	4.49 (1.76-11.46)	0.002	81
180-day mortality	1071	1.73 (0.96-3.14)	0.07	89
Biomarker type				
Inflammatory biomarker	2187	2.44 (1.63-3.66)	< 0.001	88
Metabolic biomarker	1506	1.24 (1.13-1.35)	< 0.001	30
Enzymatic biomarker	1749	2.08 (0.93-4.67)	0.08	93
Organ functional biomarker	1392	1.73 (1.03-2.92)	0.04	90
Region of patients				
East Asia	1356	2.40 (1.76-3.27)	< 0.001	86
North America	2664	1.16 (1.02-1.31)	0.02	95
South America	110	3.10 (2.14-4.50)	–	–
Middle East	306	1.45 (1.12-1.88)	–	–

#### 3.4.1 Subgroup analysis by outcome measures

**Figure 4:**
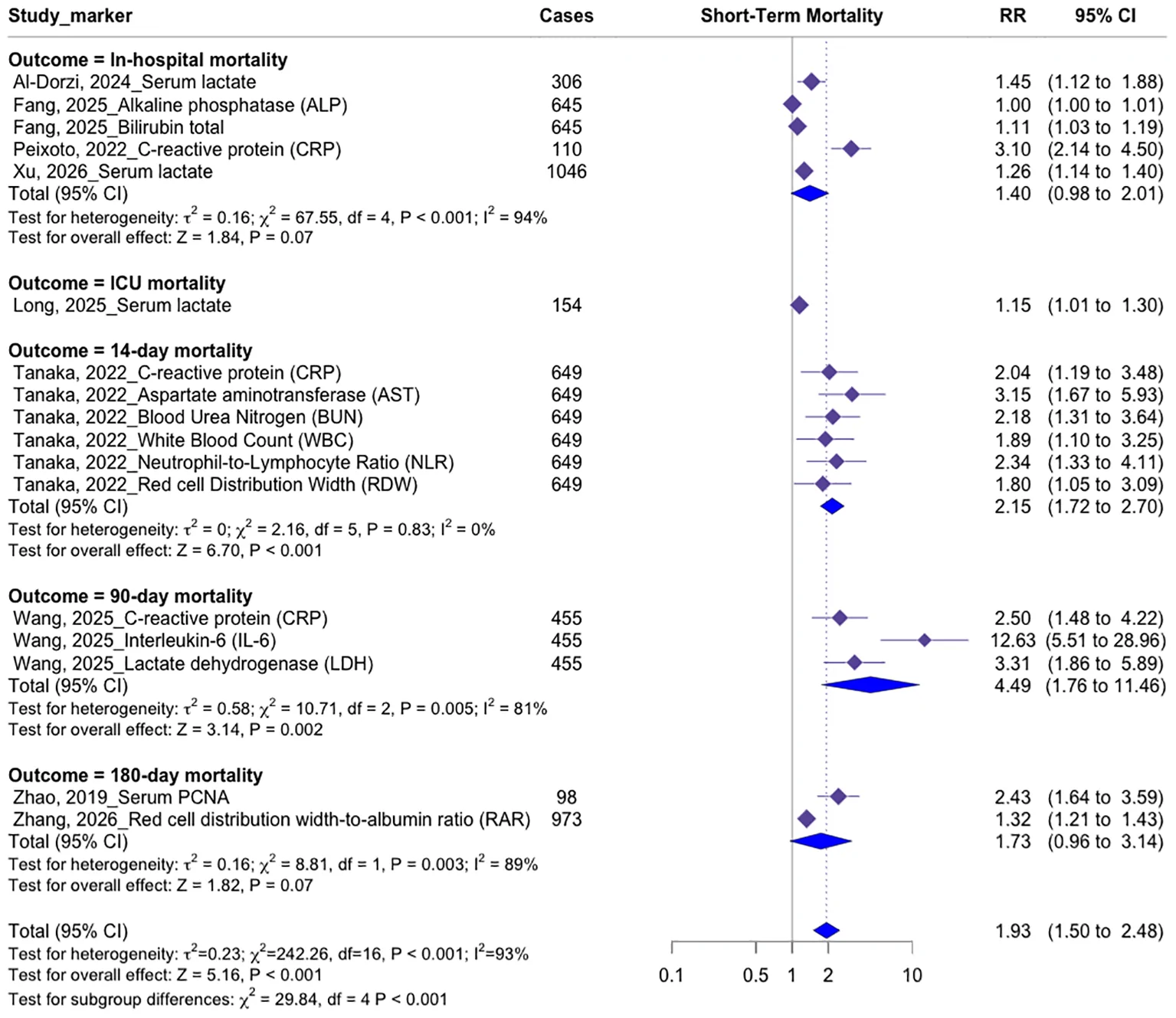
Forest plot of subgroup analysis by different outcomes.

This study conducted subgroup analysis according to different outcome measures. Results showed: in-hospital mortality subgroup (5 studies, 2,107 cases) pooled RR = 1.40 (95% CI: 0.98-2.01, *P* = 0.07); 14-day mortality subgroup (6 studies, 649 cases) pooled RR = 2.15 (95% CI: 1.72-2.70, *P* < 0.001); 90-day mortality subgroup (3 studies, 455 cases) pooled RR = 4.49 (95% CI: 1.76-11.46, *P* = 0.002); 180-day mortality subgroup (2 studies, 1,071 cases) pooled RR = 1.73 (95% CI: 0.96-3.14, *P* = 0.07) (**Table 2**; **Figure 4**). Test for differences between subgroups P < 0.001 (**Figure 4**), indicating significant differences between different outcome measure subgroups. Outcome subgroup analysis results showed different pooled RR values for different outcomes, with 90-day mortality having the highest pooled RR value, followed by 14-day mortality. This difference may reflect different death mechanisms at different time points, with early deaths more related to acute complications, while late deaths more related to tumor progression.

#### 3.4.2 Subgroup analysis by biomarker type

Different types of biomarkers have different pathophysiological mechanisms. To evaluate which biomarker types have higher predictive value for short-term mortality, this study categorized biomarker types into 4 subgroups based on biological function and clinical significance: (1) Inflammatory biomarker subgroup: including all inflammatory types (8 studies); (2) Metabolic biomarker subgroup: containing metabolic biomarkers (3 studies); (3) Enzymatic biomarker subgroup: enzymatic biomarkers (3 studies); (4) Organ functional biomarker subgroup: hepatic/functional, renal/functional, and cellular proliferation (3 studies).

**Figure 5:**
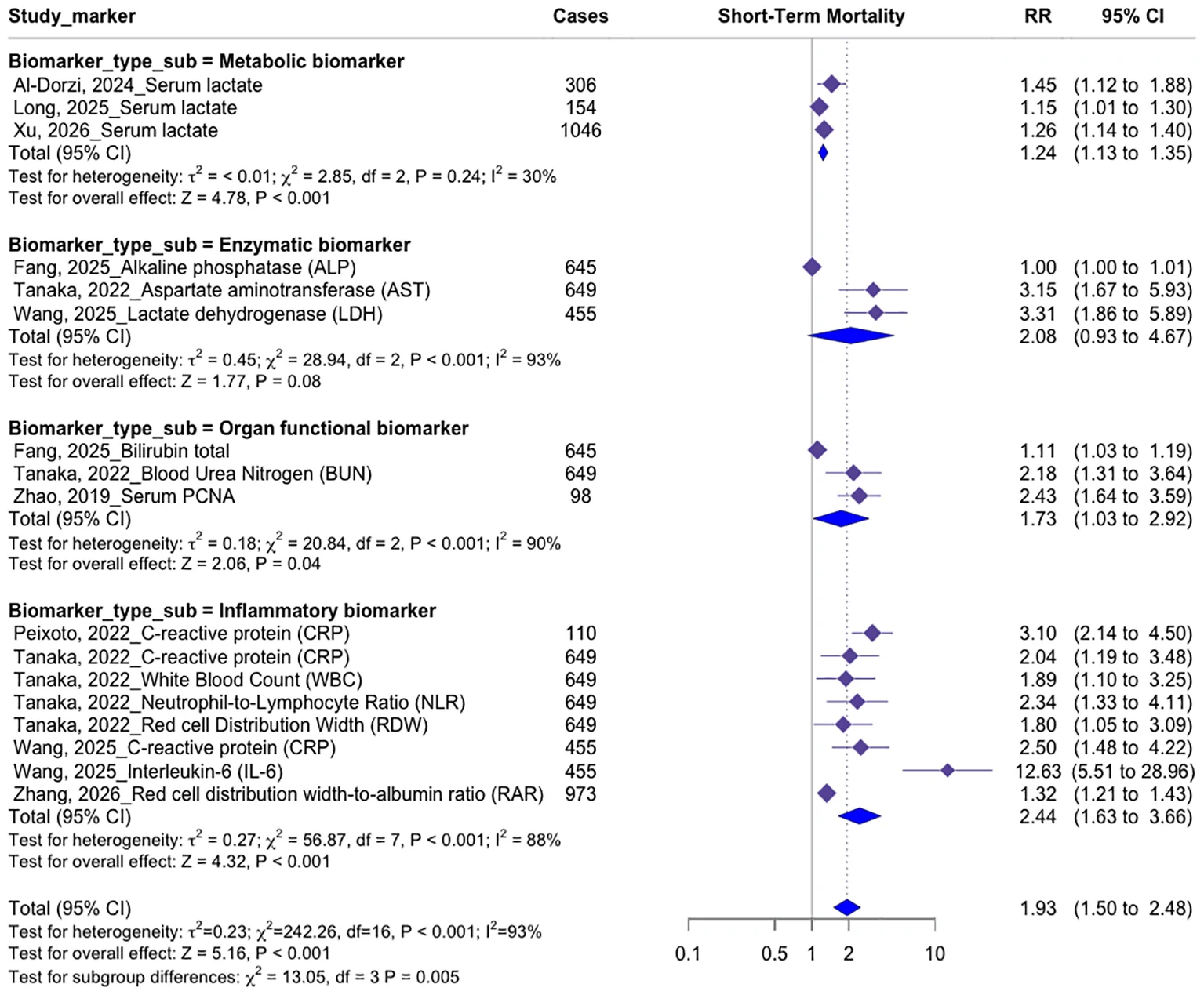
Forest plot of subgroup analysis by biomarker type.

Subgroup analysis by different biomarker types showed: inflammatory biomarker subgroup (8 studies, 2,187 cases) pooled RR = 2.44 (95% CI: 1.63-3.66, *P* < 0.001); metabolic biomarker subgroup (3 studies, 1,506 cases) pooled RR = 1.24 (95% CI: 1.13-1.35, *P* < 0.001); enzymatic biomarker subgroup (3 studies, 1,749 cases) pooled RR = 2.08 (95% CI: 0.93-4.67, *P* = 0.08); organ functional biomarker subgroup (3 studies, 1,392 cases) pooled RR = 1.73 (95% CI: 1.03-2.92, *P* = 0.04) (**Table 2**; **Figure 5**). Test for differences between biomarker type subgroups P = 0.005 (**Figure 5**), indicating significant differences between different biomarker types. The inflammatory markers subgroup had the highest pooled RR value, followed by the enzymatic biomarker subgroup. Inflammatory markers represented by CRP, IL-6, NLR, and RDW showed the largest effect sizes, suggesting that systemic inflammatory response plays a central role in short-term prognosis of critically ill lung cancer patients. Additionally, subgroup analysis found that metabolic marker serum lactate had a small but stable effect size (*I²* = 30%).

#### 3.4.3 Subgroup analysis by patient region

**Figure 6:**
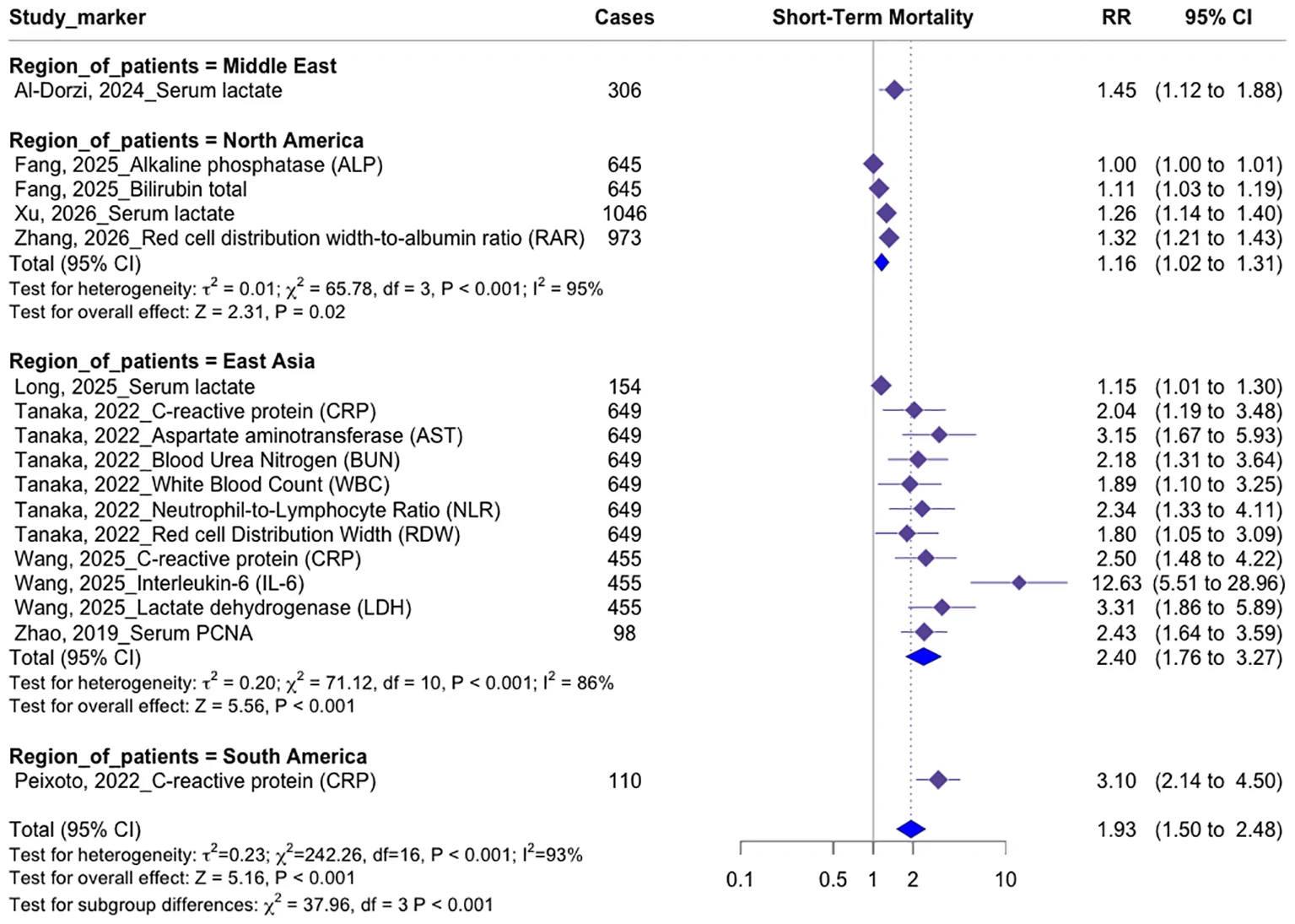
Forest plot of subgroup analysis by patient region.

To assess regional differences, this study conducted subgroup analysis by patient geographical regions. Results showed: East Asia subgroup (11 studies, 1,356 cases) pooled RR = 2.40 (95% CI: 1.76-3.27, *P* < 0.001); North America subgroup (4 studies, 2,664 cases) pooled RR = 1.16 (95% CI: 1.02-1.31, *P* = 0.02) (**Table 2**; **Figure 6**). Test for differences between subgroups *P* < 0.001 (**Figure 6**), indicating significant differences between different geographical regions. This suggests possible influences of genetic background, medical level, and environmental factors, with higher heterogeneity in North American region patients.

### 3.5 Meta-regression analysis

To explore sources of heterogeneity in the relationship between elevated biomarkers and patient short-term mortality and identify potential influencing factors, this study further conducted meta-regression analysis. Meta-regression analysis results are shown in scatter plots (**Supplementary Figures 1-4**) and coefficient table (**Table 3**).

**Table 3:** Univariate and multivariate meta-regression analysis results.

Variables	Univariate meta regression			Multivariate meta regression		
	*β*	SE	*P* value	*β*	SE	*P* value
In-hospital mortality (Outcome)	-0.445	0.248	0.048			
90-day mortality (Outcome)	0.645	0.333	0.039	0.713	0.355	0.044
North America (Region of patients)	-0.695	0.216	0.001			

Univariate meta-regression analysis showed that two outcome measures (in-hospital mortality and 90-day mortality) were significantly correlated with RR (β = -0.445, SE = 0.248, *P* = 0.048; β = 0.645, SE = 0.333, *P* = 0.039), additionally, patient region (North America) was also significantly correlated with effect size RR (β = -0.695, SE = 0.216, *P* = 0.001); multivariate meta-regression analysis showed that outcome measure (90-day mortality) was significantly correlated with effect size RR (β = 0.713, SE = 0.355, *P* = 0.044). This indicates that outcome measure (90-day mortality) can partially explain the heterogeneity of meta-analysis results. Sample size and biomarker types did not show significant effects in meta-regression analysis (*P* > 0.05), suggesting that the main sources of heterogeneity are outcome measures and patient regional differences.

### 3.6 Sensitivity analysis

**Figure 7:**
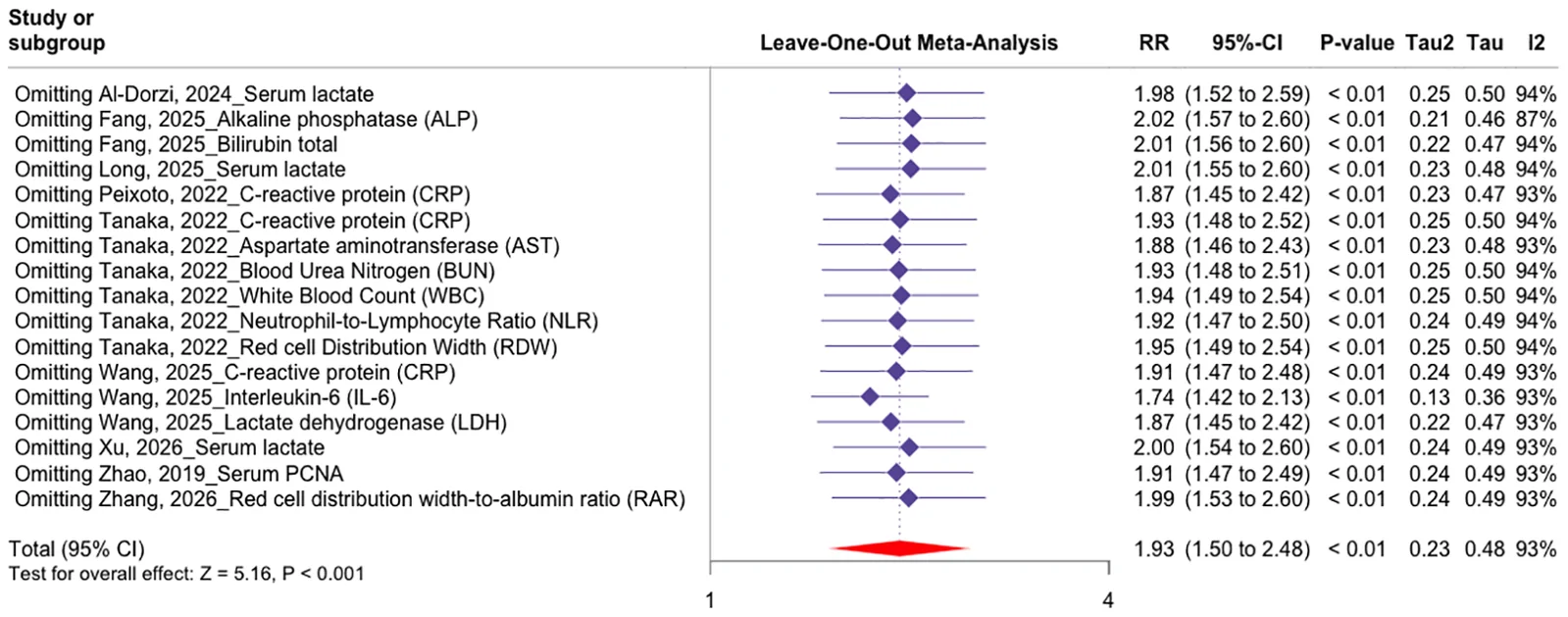
Forest plot of leave-one-out sensitivity analysis.

This study conducted sensitivity analysis to assess the robustness of meta-analysis results. The leave-one-out forest plot shows pooled RR values after sequentially excluding each study. After sequentially excluding each study, no single included study significantly affected the pooled RR value, with RR ranging from 1.74 (95% CI: 1.42-2.13) to 2.02 (95% CI: 1.57-2.60), and all RR 95% confidence intervals contained the original pooled RR value with consistent direction (**Figure 7**), indicating the stability of this study’s meta-analysis results.

Egger’s test (P = 0.18) and Begg’s test (P = 0.31) showed no evidence of significant publication bias. The trim-and-fill method did not impute any missing studies, further supporting the absence of publication bias.

## 4 Discussion

This meta-analysis systematically evaluated the association between multiple markers and short-term mortality in critically ill lung cancer patients, with main findings including: (1) Overall, elevated markers were significantly associated with short-term mortality (pooled RR = 1.66, 95% CI: 1.18-2.34, *P* < 0.001), but with high heterogeneity between studies; (2) The majority of studies (17/19) showed positive associations, with pooled RR = 1.93 (95% CI: 1.50-2.48, *P* < 0.001), serum albumin and PDCD5 were protective factors for short-term prognosis in critically ill lung cancer patients, while other markers were risk factors for short-term prognosis; (3) Subgroup analysis showed that inflammatory biomarkers (RR = 2.44) and 90-day mortality (RR = 4.49) had the largest effect sizes, with outcome measures, marker types, and geographical regions being important sources of heterogeneity; (4) Subgroup analysis by critical illness definition showed a significantly larger effect in patients with advanced/terminal disease (RR = 2.45) compared to ICU-admitted patients (RR = 1.38); (5) Sensitivity analysis supported the robustness of this study’s meta-analysis results. This study confirmed the value of multiple biomarkers in prognostic assessment of critically ill lung cancer patients.

The high heterogeneity (I² = 94%) observed in this meta-analysis warrants careful consideration. While the random-effects model and RVE provide appropriate statistical frameworks for pooling such data, the substantial variability across studies suggests that the pooled estimate represents an average effect that may not be uniformly applicable. The heterogeneity likely reflects genuine clinical and methodological diversity, including differences in biomarker types, outcome definitions, patient populations, and healthcare settings. Rather than undermining the validity of the findings, this heterogeneity highlights the complexity of prognostic assessment in this patient population and underscores the need for standardized approaches in future research.

This study’s results are consistent with previous research. Al-Dorzi et al. (17) found that ICU mortality significantly decreased after 2015, but critical care scores remained important predictors. Soares et al. (26) emphasized that organ failure and cancer progression are important death factors, consistent with this study’s findings on organ function markers’ value. Koch et al. (27) proposed that tumor-specific variables and acute disease severity jointly determine prognosis, supporting this study’s approach of combined multi-marker assessment. This study’s results are basically consistent with previous meta-analyses targeting general lung cancer patients, but with larger effect sizes. For example, a meta-analysis targeting non-small cell lung cancer patients showed that elevated pre-treatment CRP was associated with shortened overall survival (HR = 1.90, 95% CI: 1.50-2.30) (28), while inflammatory markers in this study had RR of 2.44 for short-term death, suggesting that the prognostic impact of inflammatory response is amplified in critical states. Notably, this study found that elevated albumin has a protective effect (RR = 0.28), consistent with traditional understanding of albumin as a nutritional and inflammation-negative correlation indicator.

Mechanistically, the prognostic value of multiple types of biomarkers in critically ill patients may involve several interconnected pathways: (1) Inflammation-immune mechanism: Lung cancer patients often have chronic inflammatory states, where pro-inflammatory factors such as CRP and IL-6 can activate the NF-κB pathway, promoting tumor progression and immune escape. In critical environments, inflammatory cascades can induce sepsis and acute respiratory distress syndrome (ARDS), significantly increasing short-term death risk (29). (2) Metabolism-hypoxia mechanism: Critically ill lung cancer patients often exist in tissue hypoxia states due to ventilation dysfunction or tumor embolism, with increased anaerobic glycolysis leading to lactate accumulation. Hyperlactatemia not only reflects tissue perfusion insufficiency but can also inhibit immune cell function, forming a vicious cycle (30). (3) Organ function reserve mechanism: Elevated markers such as ALP, BUN, and bilirubin indicate liver and kidney dysfunction, reflecting damage to multiple organs from tumor infiltration, drug toxicity, or systemic inflammation (31). Albumin, as a negative acute-phase reaction protein, its decreased levels are dually related to poor nutritional status and high inflammatory load (32). (4) Cell proliferation and apoptosis mechanism: Elevated PCNA reflects high tumor proliferative activity, while elevated PDCD5 may represent enhanced tumor cell apoptosis, with their different effects on prognosis consistent with their biological functions (22).

This meta-analysis is the first to systematically compare the predictive performance of four types of biomarkers in critically ill lung cancer patients. Inflammatory markers (CRP, IL-6, NLR, and RDW) showed the largest effect sizes, suggesting that tumor-related systemic inflammatory response is the core mechanism driving short-term death. Metabolic markers, such as serum lactate, had smaller but low-heterogeneity effect sizes, indicating their stable predictive value as hypoxia indicators. Enzymatic markers (ALP, AST, and LDH) had moderate but high-heterogeneity effect sizes, possibly related to tumor burden and liver metastasis. Organ function markers (BUN, bilirubin, PCNA) also showed positive effect sizes, reflecting the cumulative effect of multiple organ dysfunction. Therefore, combined detection of multiple types of markers may provide more comprehensive risk stratification information.

The prognostic value of multiple types of markers in critically ill patients may involve multiple mechanisms: (1) Inflammation-immune mechanism: Lung cancer patients often have chronic inflammatory states, where pro-inflammatory factors such as CRP and IL-6 can activate the NF-κB pathway, promoting tumor progression and immune escape. In critical environments, inflammatory cascades can induce sepsis and acute respiratory distress syndrome (ARDS), significantly increasing short-term death risk (29). (2) Metabolism-hypoxia mechanism: Critically ill lung cancer patients often exist in tissue hypoxia states due to ventilation dysfunction or tumor embolism, with increased anaerobic glycolysis leading to lactate accumulation. Hyperlactatemia not only reflects tissue perfusion insufficiency but can also inhibit immune cell function, forming a vicious cycle (30). (3) Organ function reserve mechanism: Elevated markers such as ALP, BUN, and bilirubin indicate liver and kidney dysfunction, reflecting damage to multiple organs from tumor infiltration, drug toxicity, or systemic inflammation (31). Albumin, as a negative acute-phase reaction protein, its decreased levels are dually related to poor nutritional status and high inflammatory load (32). (4) Cell proliferation and apoptosis mechanism: Elevated PCNA reflects high tumor proliferative activity, while elevated PDCD5 may represent enhanced tumor cell apoptosis, with their different effects on prognosis consistent with their biological functions (22).

Based on this study’s meta-analysis results, the following clinical application recommendations can be made: (1) Upon admission of critically ill lung cancer patients, routine detection of inflammatory markers (CRP, NLR), metabolic markers (lactate, albumin), and enzymatic markers (LDH) should be performed; (2) Integration of biomarkers into traditional critical care scoring systems, such as APACHE II-SOFA combined scoring, to establish multi-marker-based risk assessment models, may improve predictive accuracy; (3) For patients with significantly elevated markers, more active infection control, shock correction, and nutritional support should be provided. This study’s results will provide important evidence-based medical evidence for prognostic assessment of critically ill lung cancer patients. Biomarkers, as simple, economical, and repeatable detection indicators, have important practical value in critical environments. Compared with complex critical care scoring systems, marker detection has advantages of being rapid, simple, and low-cost, more suitable for rapid assessment in emergency and early critical care phases.

This meta-analysis has the following strengths: (1) This study is the first meta-analysis to systematically evaluate the association between multiple types of biomarkers and short-term mortality in critically ill lung cancer patients; (2) This study included 4,436 patients with large sample size and sufficient statistical power; (3) This study’s subgroup analysis was comprehensive, analyzing by outcome measures, marker types, and patient geographical regions to deeply explore sources of heterogeneity; (4) This study’s methodology was rigorous, following PRISMA guidelines and using multiple methods including sensitivity analysis and meta-regression to ensure result robustness; (5) This study has high clinical translation value, with the proposed marker combinations and risk stratification strategies being easy to implement in ICU environments.

This meta-analysis has the following limitations: (1) All included studies were retrospective cohort studies, with risks of selection bias and information bias, and limited causal inference ability; (2) Very high heterogeneity (I² = 94%), although some sources were explored through subgroup analysis and meta-regression, residual heterogeneity still exists, including different biomarker cutoff values used in various studies, inconsistent critical illness definition standards, and inability to stratify detailed differences in lung cancer pathological types and stages; the pooled estimate should therefore be interpreted as an average effect and applied with caution in individual clinical settings; (3) Uneven geographical distribution of patients, with most studies from East Asia (Japan, China) and North America (MIMIC database), only 1 study each from South America and Middle East, requiring caution when extrapolating this study’s results to Africa, Europe, and other regions; (4) The study limited inclusion to English and Chinese publications, potentially introducing language bias; (5) The conversion of ORs and HRs to RRs required assumptions about baseline risk and proportional hazards, which may introduce additional uncertainty; (6) Lack of individual patient data, preventing time-to-event analysis and adjustment for confounding factors; (7) The inclusion of two studies with protective effects (albumin, PDCD5) in a meta-analysis primarily examining risk factors represents a methodological limitation, as combining risk and protective factors in a single pooled estimate may be conceptually problematic; we addressed this by presenting these separately in **Figure 3** and in the text.

Based on this meta-analysis’s findings and limitations, future research should focus on the following directions: (1) Conduct prospective cohort studies with unified design, standardizing marker detection methods and cutoff values, collecting complete TNM staging, treatment history, gene mutation status, and other data to validate this study’s meta-analysis conclusions; (2) Develop and validate prognostic models integrating biomarkers with traditional scores such as APACHE II and SOFA to evaluate their prognostic value; (3) Design randomized controlled trials to evaluate whether enhanced treatment strategies based on elevated markers, such as early anti-inflammatory therapy, nutritional support, and renal replacement therapy, can improve patient survival; (4) Combine proteomics and metabolomics technologies to discover new prognostic biomarkers specific to critically ill lung cancer; (5) Use machine learning methods to process marker data and construct individualized risk prediction tools.

## 5 Conclusion

This meta-analysis confirmed that elevated multiple biomarkers are significantly associated with short-term mortality in critically ill lung cancer patients, with main conclusions including: (1) Overall, elevated markers are independent risk factors for short-term mortality in critically ill lung cancer patients, increasing short-term death risk by approximately 66% (RR = 1.66); (2) Inflammatory biomarkers CRP, IL-6, NLR, and RDW have the most prominent predictive performance (RR = 2.44); (3) 90-day mortality is the outcome measure with the largest effect size (RR = 4.49); (4) Serum albumin and PDCD5 are independent protective factors for short-term prognosis in critically ill lung cancer patients; (5) Significant differences exist between different patient geographical regions, such as East Asia vs. North America significantly affecting effect sizes, suggesting modifying effects of genetic and environmental factors. (6) The definition of critical illness (ICU admission vs. advanced/terminal disease) significantly modifies the association, with stronger effects observed in patients with advanced disease. These findings from this study provide important evidence-based medical evidence for risk stratification, treatment decision-making, and prognostic assessment in critically ill lung cancer patients. However, the high heterogeneity across studies and the retrospective nature of all included evidence indicate that these results should be interpreted with caution. The pooled estimates provide a useful summary of the current evidence but may not be directly applicable to all clinical settings or individual patients. Future research should conduct prospective large-sample studies to validate this study’s results, explore the combined application of biomarkers with traditional prognostic scoring systems, study the predictive value of biomarker dynamic changes on prognosis, and develop individualized prognostic prediction models based on circulating biomarkers. In clinical practice, it is recommended to include biomarker detection in routine assessment of critically ill lung cancer patients, combining with clinical characteristics and traditional scoring systems to establish comprehensive risk assessment systems and provide evidence for individualized treatment.

## Data Availability

The raw data supporting the conclusions of this article will be made available by the authors, without undue reservation.
