# Supplementary material for: Association between elevated multiple circulating biomarkers and short-term mortality in critically ill lung cancer patients: a meta-analysis
Source: Front Oncol. 2026 Aug 19;16:1861784. doi: 10.3389/fonc.2026.1861784 (PMC13533784; doi:10.3389/fonc.2026.1861784)
Supplement: Supplementary file 1 [file Table1.docx]

**Table S1. Detailed Search Strategies for Each Database**

| **Database** | **Search Strategy** | **Results** |
| --- | --- | --- |
| **PubMed** | ("Lung Neoplasms"[Mesh] OR "lung cancer"[tiab] OR "lung neoplasm"[tiab] OR "pulmonary carcinoma"[tiab] OR "non-small cell lung cancer"[tiab] OR "NSCLC"[tiab] OR "small cell lung cancer"[tiab] OR "SCLC"[tiab]) AND ("Critical Care"[Mesh] OR "Intensive Care Units"[Mesh] OR "critical care"[tiab] OR "intensive care"[tiab] OR "ICU"[tiab] OR "critically ill"[tiab] OR "critical illness"[tiab]) AND ("Biomarkers, Tumor"[Mesh] OR "biomarker"[tiab] OR "tumor marker"[tiab] OR "CEA"[tiab] OR "carcinoembryonic antigen"[tiab] OR "CYFRA21-1"[tiab] OR "NSE"[tiab] OR "CA125"[tiab] OR "CRP"[tiab] OR "C-reactive protein"[tiab] OR "NLR"[tiab] OR "neutrophil-to-lymphocyte ratio"[tiab] OR "PLR"[tiab] OR "platelet-to-lymphocyte ratio"[tiab] OR "IL-6"[tiab] OR "interleukin-6"[tiab] OR "lactate"[tiab] OR "albumin"[tiab] OR "LDH"[tiab] OR "lactate dehydrogenase"[tiab] OR "AST"[tiab] OR "aspartate aminotransferase"[tiab] OR "ALP"[tiab] OR "alkaline phosphatase"[tiab] OR "BUN"[tiab] OR "blood urea nitrogen"[tiab] OR "bilirubin"[tiab] OR "RDW"[tiab] OR "red cell distribution width"[tiab] OR "PCNA"[tiab] OR "PDCD5"[tiab]) AND ("Mortality"[Mesh] OR "mortality"[tiab] OR "death"[tiab] OR "survival"[tiab] OR "prognosis"[tiab] OR "short-term"[tiab] OR "30-day"[tiab] OR "in-hospital"[tiab] OR "ICU mortality"[tiab] OR "90-day"[tiab] OR "180-day"[tiab]) | **2,847** |
| **Embase** | ('lung cancer'/exp OR 'lung cancer':ti,ab OR 'lung neoplasm':ti,ab OR 'pulmonary carcinoma':ti,ab OR 'non-small cell lung cancer':ti,ab OR 'nsclc':ti,ab OR 'small cell lung cancer':ti,ab OR 'sclc':ti,ab) AND ('intensive care'/exp OR 'critical care':ti,ab OR 'intensive care':ti,ab OR 'icu':ti,ab OR 'critically ill':ti,ab OR 'critical illness':ti,ab) AND ('tumor marker'/exp OR 'biomarker':ti,ab OR 'tumor marker':ti,ab OR 'cea':ti,ab OR 'carcinoembryonic antigen':ti,ab OR 'cyfra21-1':ti,ab OR 'nse':ti,ab OR 'ca125':ti,ab OR 'crp':ti,ab OR 'c-reactive protein':ti,ab OR 'nlr':ti,ab OR 'neutrophil-to-lymphocyte ratio':ti,ab OR 'plr':ti,ab OR 'platelet-to-lymphocyte ratio':ti,ab OR 'il-6':ti,ab OR 'interleukin-6':ti,ab OR 'lactate':ti,ab OR 'albumin':ti,ab OR 'ldh':ti,ab OR 'lactate dehydrogenase':ti,ab OR 'ast':ti,ab OR 'aspartate aminotransferase':ti,ab OR 'alp':ti,ab OR 'alkaline phosphatase':ti,ab OR 'bun':ti,ab OR 'blood urea nitrogen':ti,ab OR 'bilirubin':ti,ab OR 'rdw':ti,ab OR 'red cell distribution width':ti,ab OR 'pcna':ti,ab OR 'pdcd5':ti,ab) AND ('mortality'/exp OR 'mortality':ti,ab OR 'death':ti,ab OR 'survival':ti,ab OR 'prognosis':ti,ab OR 'short-term':ti,ab OR '30-day':ti,ab OR 'in-hospital':ti,ab OR 'icu mortality':ti,ab OR '90-day':ti,ab OR '180-day':ti,ab) | **3,124** |
| **Web of Science** | TS=("lung cancer" OR "lung neoplasm" OR "pulmonary carcinoma" OR "non-small cell lung cancer" OR "NSCLC" OR "small cell lung cancer" OR "SCLC") AND TS=("critical care" OR "intensive care" OR "ICU" OR "critically ill" OR "critical illness") AND TS=("biomarker" OR "tumor marker" OR "CEA" OR "carcinoembryonic antigen" OR "CYFRA21-1" OR "NSE" OR "CA125" OR "CRP" OR "C-reactive protein" OR "NLR" OR "neutrophil-to-lymphocyte ratio" OR "PLR" OR "platelet-to-lymphocyte ratio" OR "IL-6" OR "interleukin-6" OR "lactate" OR "albumin" OR "LDH" OR "lactate dehydrogenase" OR "AST" OR "aspartate aminotransferase" OR "ALP" OR "alkaline phosphatase" OR "BUN" OR "blood urea nitrogen" OR "bilirubin" OR "RDW" OR "red cell distribution width" OR "PCNA" OR "PDCD5") AND TS=("mortality" OR "death" OR "survival" OR "prognosis" OR "short-term" OR "30-day" OR "in-hospital" OR "ICU mortality" OR "90-day" OR "180-day") | **1,055** |
| **Cochrane Library** | ("lung cancer" OR "lung neoplasm" OR "pulmonary carcinoma" OR "non-small cell lung cancer" OR "NSCLC" OR "small cell lung cancer" OR "SCLC") AND ("critical care" OR "intensive care" OR "ICU" OR "critically ill" OR "critical illness") AND ("biomarker" OR "tumor marker" OR "CEA" OR "CRP" OR "NLR" OR "lactate" OR "albumin" OR "LDH" OR "bilirubin" OR "RDW") AND ("mortality" OR "death" OR "survival" OR "prognosis" OR "short-term") | **0** |
| **CNKI (China National Knowledge Infrastructure)** | (SU='肺癌' OR SU='肺恶性肿瘤' OR SU='非小细胞肺癌' OR SU='小细胞肺癌') AND (SU='重症监护' OR SU='ICU' OR SU='危重症' OR SU='危重患者' OR SU='重症医学') AND (SU='肿瘤标志物' OR SU='生物标志物' OR SU='CEA' OR SU='癌胚抗原' OR SU='CYFRA21-1' OR SU='NSE' OR SU='CA125' OR SU='CRP' OR SU='C反应蛋白' OR SU='NLR' OR SU='中性粒细胞淋巴细胞比值' OR SU='PLR' OR SU='血小板淋巴细胞比值' OR SU='IL-6' OR SU='白细胞介素-6' OR SU='乳酸' OR SU='白蛋白' OR SU='LDH' OR SU='乳酸脱氢酶' OR SU='AST' OR SU='谷草转氨酶' OR SU='ALP' OR SU='碱性磷酸酶' OR SU='BUN' OR SU='尿素氮' OR SU='胆红素' OR SU='RDW' OR SU='红细胞分布宽度') AND (SU='死亡率' OR SU='病死率' OR SU='预后' OR SU='生存' OR SU='短期' OR SU='住院期间' OR SU='30天' OR SU='90天') | **0** |
| **Additional records identified through reference searching** | Manual screening of reference lists of included studies and relevant review articles | **329** |
| **TOTAL** |  | **7,355** |
