# Supplementary material for: Association between elevated multiple circulating biomarkers and short-term mortality in critically ill lung cancer patients: a meta-analysis
Source: Front Oncol. 2026 Aug 19;16:1861784. doi: 10.3389/fonc.2026.1861784 (PMC13533784; doi:10.3389/fonc.2026.1861784)
Supplement: Supplementary file 2 [file Table2.docx]

**Table S2. Newcastle-Ottawa Scale (NOS) Quality Assessment Details**

| **Study** | **Selection (Max 4)** |  |  |  | **Comparability (Max 2)** | **Outcome (Max 3)** |  |  | **Total Score (Max 9)** |
| --- | --- | --- | --- | --- | --- | --- | --- | --- | --- |
|  | 1. Adequate definition of cases | 2. Representativeness of cases | 3. Selection of controls | 4. Definition of controls | 5. Comparability of cohorts based on design/analysis | 6. Assessment of outcome | 7. Adequate follow-up duration | 8. Adequacy of follow-up |  |
| Al-Dorzi, 2024 | 1 | 1 | 1 | 1 | 1 | 1 | 1 | 1 | 8 |
| Fang, 2025a | 1 | 1 | 1 | 1 | 1 | 1 | 1 | 1 | 8 |
| Fang, 2025b | 1 | 1 | 1 | 1 | 1 | 1 | 1 | 1 | 8 |
| Long, 2025 | 1 | 1 | 1 | 0 | 1 | 1 | 1 | 1 | 7 |
| Peixoto, 2022 | 1 | 1 | 1 | 0 | 1 | 1 | 1 | 1 | 7 |
| Tanaka, 2022a | 1 | 1 | 1 | 1 | 1 | 1 | 1 | 1 | 8 |
| Tanaka, 2022b | 1 | 1 | 1 | 1 | 1 | 1 | 1 | 1 | 8 |
| Tanaka, 2022c | 1 | 1 | 1 | 1 | 1 | 1 | 1 | 1 | 8 |
| Tanaka, 2022d | 1 | 1 | 1 | 1 | 1 | 1 | 1 | 1 | 8 |
| Tanaka, 2022e | 1 | 1 | 1 | 1 | 1 | 1 | 1 | 1 | 8 |
| Tanaka, 2022f | 1 | 1 | 1 | 1 | 1 | 1 | 1 | 1 | 8 |
| Wang, 2025a | 1 | 1 | 1 | 1 | 1 | 1 | 1 | 1 | 8 |
| Wang, 2025b | 1 | 1 | 1 | 1 | 1 | 1 | 1 | 1 | 8 |
| Wang, 2025c | 1 | 1 | 1 | 1 | 1 | 1 | 1 | 1 | 8 |
| Wang, 2025d | 1 | 1 | 1 | 1 | 1 | 1 | 1 | 1 | 8 |
| Xu, 2026 | 1 | 1 | 1 | 1 | 1 | 1 | 1 | 1 | 8 |
| Zhao, 2019a | 1 | 1 | 1 | 0 | 1 | 1 | 1 | 1 | 7 |
| Zhao, 2019b | 1 | 1 | 1 | 0 | 1 | 1 | 1 | 1 | 7 |
| Zhang, 2026 | 1 | 1 | 1 | 1 | 1 | 1 | 1 | 1 | 8 |

**NOS Scoring Criteria Explanation:**

1. **Adequate definition of cases:** Yes = 1 point (pathologically confirmed lung cancer); No = 0 points
2. **Representativeness of cases:** Yes = 1 point (consecutive or all eligible cases); No = 0 points
3. **Selection of controls:** Yes = 1 point (controls selected from same population); No = 0 points (not applicable for cohort studies, automatically scored 1)
4. **Definition of controls:** Yes = 1 point (controls have no history of outcome); No = 0 points (not applicable for cohort studies, automatically scored 1)
5. **Comparability based on design or analysis:** Yes = 1 point for controlling for age/sex, additional point for controlling for other relevant confounders (maximum 2 points)

**Outcome (3 items, 1 point each):**

6. **Assessment of outcome:** Yes = 1 point (independent blind assessment or record linkage); No = 0 points
7. **Adequate follow-up duration:** Yes = 1 point (sufficient time for outcome to occur); No = 0 points
8. **Adequacy of follow-up:** Yes = 1 point (complete follow-up or loss ≤20%); No = 0 points

**Quality Categories:**

- **High quality:** 7-9 points
- **Moderate quality:** 4-6 points
- **Low quality:** 0-3 points
