# Supplementary material for: Association between elevated multiple circulating biomarkers and short-term mortality in critically ill lung cancer patients: a meta-analysis
Source: Front Oncol. 2026 Aug 19;16:1861784. doi: 10.3389/fonc.2026.1861784 (PMC13533784; doi:10.3389/fonc.2026.1861784)
Supplement: Supplementary file 3 [file Table3.docx]

**Table S3. Units of Measurement, Cut-off Values, and Statistical Methods for Each Biomarker in Included Studies**

| **Study** | **Biomarker** | **Biomarker Type** | **Unit of Measurement** | **Cut-off Value** | **Statistical Method** | **Effect Size Reported** |
| --- | --- | --- | --- | --- | --- | --- |
| Al-Dorzi, 2024 | Serum lactate | Metabolic biomarker | mmol/L | ≥2.0 mmol/L | Multivariate logistic regression | OR (95% CI) |
| Fang, 2025a | Alkaline phosphatase (ALP) | Enzymatic biomarker | U/L | ≥104 U/L | Multivariate Cox regression | HR (95% CI) |
| Fang, 2025b | Bilirubin total | Hepatic/functional biomarker | mg/dL | ≥1.2 mg/dL | Multivariate Cox regression | HR (95% CI) |
| Long, 2025 | Serum lactate | Metabolic biomarker | mmol/L | ≥2.0 mmol/L | Multivariate logistic regression | OR (95% CI) |
| Peixoto, 2022 | C-reactive protein (CRP) | Inflammatory biomarker | mg/L | ≥10 mg/L | Multivariate logistic regression | OR (95% CI) |
| Tanaka, 2022a | C-reactive protein (CRP) | Inflammatory biomarker | mg/dL | ≥1.0 mg/dL | Multivariate logistic regression | OR (95% CI) |
| Tanaka, 2022b | Aspartate aminotransferase (AST) | Enzymatic biomarker | U/L | ≥40 U/L | Multivariate logistic regression | OR (95% CI) |
| Tanaka, 2022c | Blood Urea Nitrogen (BUN) | Renal/functional biomarker | mg/dL | ≥20 mg/dL | Multivariate logistic regression | OR (95% CI) |
| Tanaka, 2022d | White Blood Count (WBC) | Hematologic/inflammatory biomarker | ×10⁹/L | ≥10,000/μL | Multivariate logistic regression | OR (95% CI) |
| Tanaka, 2022e | Neutrophil-to-Lymphocyte Ratio (NLR) | Hematologic/inflammatory biomarker | Ratio | ≥5.0 | Multivariate logistic regression | OR (95% CI) |
| Tanaka, 2022f | Red cell Distribution Width (RDW) | Hematologic/inflammatory biomarker | % | ≥15% | Multivariate logistic regression | OR (95% CI) |
| Wang, 2025a | C-reactive protein (CRP) | Inflammatory biomarker | mg/L | >10 mg/L | Multivariate Cox regression | HR (95% CI) |
| Wang, 2025b | Interleukin-6 (IL-6) | Inflammatory biomarker | pg/mL | >7 pg/mL | Multivariate Cox regression | HR (95% CI) |
| Wang, 2025c | Serum albumin (ALB) | Nutritional/hepatic synthetic biomarker | g/L | ≤35 g/L | Multivariate Cox regression | HR (95% CI) |
| Wang, 2025d | Lactate dehydrogenase (LDH) | Enzymatic biomarker | U/L | ≥250 U/L | Multivariate Cox regression | HR (95% CI) |
| Xu, 2026 | Serum lactate | Metabolic biomarker | mmol/L | ≥2.0 mmol/L | Multivariate logistic regression | OR (95% CI) |
| Zhao, 2019a | Serum PCNA | Cellular proliferation biomarker | ng/mL | ≥25 ng/mL | Multivariate Cox regression | HR (95% CI) |
| Zhao, 2019b | Serum PDCD5 | Apoptotic biomarker | ng/mL | ≥15 ng/mL | Multivariate Cox regression | HR (95% CI) |
| Zhang, 2026 | Red cell distribution width-to-albumin ratio (RAR) | Inflammatory/Nutritional biomarker | Ratio | ≥0.45 | Multivariate Cox regression | HR (95% CI) |

**Abbreviations:** ALP = Alkaline phosphatase; AST = Aspartate aminotransferase; BUN = Blood Urea Nitrogen; CRP = C-reactive protein; IL-6 = Interleukin-6; LDH = Lactate dehydrogenase; NLR = Neutrophil-to-Lymphocyte Ratio; PCNA = Proliferating Cell Nuclear Antigen; PDCD5 = Programmed Cell Death 5; RAR = Red cell distribution width-to-albumin ratio; RDW = Red cell Distribution Width; WBC = White Blood
